# Transcriptome Analysis of Acute Phase Liver Graft Injury in Liver Transplantation

**DOI:** 10.3390/biomedicines6020041

**Published:** 2018-04-06

**Authors:** Nikki P. Lee, Haiyang Wu, Kevin T.P. Ng, Ruibang Luo, Tak-Wah Lam, Chung-Mau Lo, Kwan Man

**Affiliations:** 1 Department of Surgery, The University of Hong Kong, Hong Kong, China; ledodes@hku.hk (K.T.P.N.); chungmlo@hkucc.hku.hk (C.-M.L.); kwanman@hku.hk (K.M.); 2 Collaborative Innovation Center for Diagnosis and Treatment of Infectious Diseases, Zhejiang University, Hangzhou 310003, China; 3 Department of Computer Science, The University of Hong Kong, Hong Kong, China; repurpledeep@gmail.com (H.W.); rbluo2@hku.hk (R.L.); twlam@cs.hku.hk (T.-W.L.)

**Keywords:** Liver transplantation, liver graft injury, intragraft gene expression profiles, cell adhesion molecules, *CD274*, *HFE*

## Abstract

Background: Liver transplantation remains the treatment of choice for a selected group of hepatocellular carcinoma (HCC) patients. However, the long-term benefit is greatly hampered by post-transplant HCC recurrence. Our previous studies have identified liver graft injury as an acute phase event leading to post-transplant tumor recurrence. Methods: To re-examine this acute phase event at the molecular level and in an unbiased way, RNA sequencing (RNA-Seq) was performed on liver graft biopsies obtained from the transplant recipients two hours after portal vein reperfusion with an aim to capture frequently altered pathways that account for post-transplant tumor recurrence. Liver grafts from recurrent recipients (*n* = 6) were sequenced and compared with those from recipients without recurrence (*n* = 5). Results: RNA expression profiles comparison pointed to several frequently altered pathways, among which pathways related to cell adhesion molecules were the most involved. Subsequent validation using quantitative polymerase chain reaction confirmed the differential involvement of two cell adhesion molecules *HFE* (hemochromatosis) and *CD274* and their related molecules in the acute phase event. Conclusion: This whole transcriptome strategy unravels the molecular landscape of liver graft gene expression alterations, which can identify key pathways and genes that are involved in acute phase liver graft injury that may lead to post-transplant tumor recurrence.

## 1. Introduction

Hepatocellular carcinoma (HCC) is a clinically challenging liver malignancy with a nearly equal worldwide incidence and death. Among various treatments, liver transplant results in a favorable survival in a well selected patient subgroup [1]. However, this treatment unavoidably accompanies ischemia and reperfusion injury that can trigger acute phase graft injury, or even rejection [2]. Acute phase liver graft injury is a hallmark event leading to late phase HCC recurrence in liver transplantation. Our previous studies using a liver transplant animal model have revealed a deregulation of signaling pathways related to inflammation, invasion and migration in the acute phase event and their associations with late phase tumor recurrence [3,4]. Besides, various molecules, e.g., CXCL10 (C-X-C motif chemokine ligand 10), and certain cells, e.g., endothelial progenitor cells, were found capable of promoting tumor recurrence after liver transplantation in studies using relevant animal models and clinical specimens [5,6]. These studies have unequivocally demonstrated that acute phase liver graft injury is an early event leading to post-transplant tumor recurrence. A better understanding of the acute phase event is important for developing new or prophylactic treatments for post-transplant tumor recurrence.

Despite the efforts that have been devoted to the study of acute phase liver graft injury and late phase HCC recurrence in liver transplantation, the molecular mechanisms underlying these events were not fully uncovered. Here, we revisited this theme by using RNA sequencing to capture intragraft gene expression changes in acute phase liver graft injury that account for post-transplant tumor recurrence, for which some of them are known for their effects on recipient outcomes. In a liver transplant animal study, cDNA microarray results revealed a number of genes, especially those inflammatory genes, were up-regulated in liver grafts that are more prone to develop tumors [4]. On the other hand, certain genes, such as *GPx3* (glutathione peroxidase 3), experienced down-regulation [7]. Apart from the above effects, intragraft gene expression changes can also influence other post-transplant outcomes, such as graft rejection. A preferential expression of genes related to signal transduction, inflammation, and immune response was detected in liver grafts undergoing acute cellular rejection, which is a common situation leading to graft loss in a specific recipient group [8]. Overall, these prior studies have put forth the importance of studying intragraft gene expression patterns in acute phase liver graft injury and their correlation with post-transplant outcomes. The derived results can enhance the understanding of acute phase liver graft injury, for which the knowledge can improve patient management in terms of risk stratification, prophylaxis and treatment of post-transplant tumor recurrence. Our long-term goal is to improve the recipient outcomes after liver transplantation.

## 2. Experimental Section

### 2.1. Clinical specimens

HCC patients that were included in this study received their liver transplant in Queen Mary Hospital, Pokfulam, Hong Kong (March 2004 to April 2010). Written patient consents were obtained. The last follow-up date was September 2013. The clinicopathological information between HCC patients with and without HCC recurrence after liver transplantation including sex, age, type of liver transplant, Milan criteria, vascular permeation, HBsAg before liver transplantation, new TNM stage, AST level (24 h after liver transplantation), and ALT level (24 h after liver transplantation) were listed in Supplementary Table S1. Among them, both the AST and ALT level in recurrence group were significantly higher than the non-recurrence group (Supplementary Table S1). The recurrence period and recurrence sites are listed in Supplementary Table S2. Liver graft biopsies collected 2 h after portal vein reperfusion during liver transplantation were frozen immediately and stored at −80 °C. The use of clinical specimens for research was approved by the Institutional Review Board of The University of Hong Kong/Hospital Authority Hong Kong West Cluster (HKU/HA HKW IRB). 

### 2.2. RNA sequencing (RNA-Seq) and data processing

**Figure 1:**
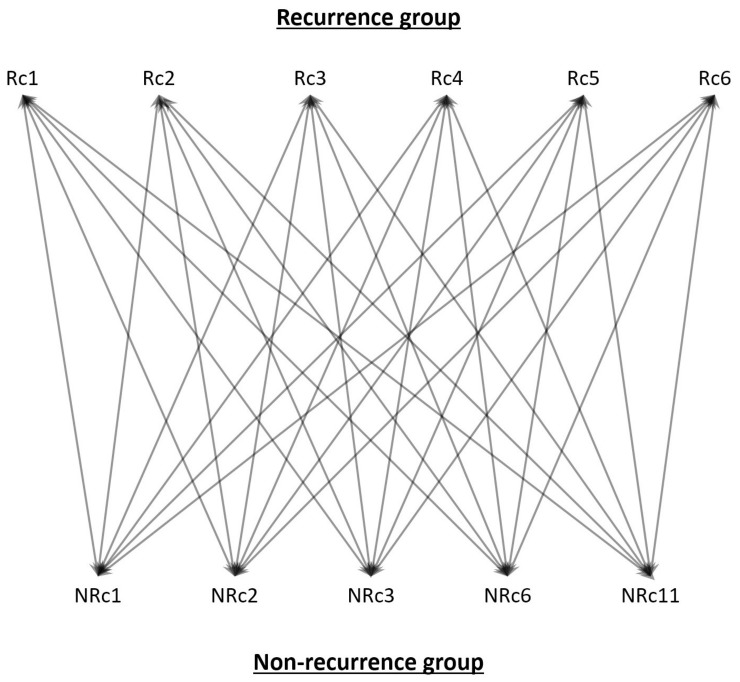
RNA-Seq data comparison method. RNA-Seq data of liver grafts from recipients with (*n* = 6) or without (*n* = 5) post-transplant hepatocellular carcinoma (HCC) recurrence was individually compared to identify differentially expressed genes, for which this result was used to map the frequently altered pathways. Rc, recurrence; NRc, non-recurrence.

RNA-Seq was performed on liver grafts from recipients with (*n* = 6) or without (*n* = 5) post-transplant HCC recurrence. The 5 patients without post-transplant HCC recurrence are patients treated with deceased donor liver transplant (DDLT). Total RNA was extracted from liver grafts using TRIzol reagent (Life Technologies, Waltham, MA, USA) as before [9,10]. RNA quality was analyzed in an Agilent 2100 Bioanalyzer (Agilent Technologies, Santa Clara, CA, USA). RNA sequencing was performed in a HiSeq 2500 System sequencer (Illumina, San Diego, CA, USA) in BGI, Hong Kong. A total of eight billion bases of sequencing data (quality control passed) was produced. The data were preprocessed using cutadapt version 1.1 to remove sequencing adapters. Then the data were mapped to the human reference genome GRCh37 (hg19) using aligner BWA version 0.6.2 with default options and then converted into BAM file format using SAMtools version 0.1.19. The gene expression level of each sample was calculated using the method introduced by Mortazavi et al. [11]. More specifically, the gene expression level difference between any given two samples was gauged by the number of sequencing data mapped to the gene, RPKM (reads per kilobase per million mapped reads). Each recurrence sample was compared to each non-recurrence sample to identify the up-regulated and down-regulated genes (Figure 1), together with their expression ratios. Using this method, a total of 30 differentially expressed gene lists were generated, which were then merged. Genes with inconsistent expression changes were removed. An average expression difference for each gene was calculated. 

### 2.3. Bioinformatics analyses

Pathway enrichment analyses were performed using Kyoto Encyclopedia of Genes and Genomes (KEGG) and Gene Ontology (GO). STRING database (http://string-db.org, version 9) was used to find related molecules for the candidate genes [12]. High confidence level at 0.700 was used.

### 2.4. Reverse transcription-quantitative polymerase chain reaction (RT-qPCR)

RT-qPCR was performed, as described [9,10]. Total RNA from liver graft biopsies were extracted as above. Reverse transcription was performed using High-Capacity cDNA Reverse Transcription kit (Applied Biosystems, Foster City, CA, USA). qPCR was performed using Power SYBR Green PCR Master Mix (Applied Biosystems) and gene-specific primers (Supplementary Table S3). *β-Actin* expression was used as an internal normalization control. The relative expression level of each gene in each sample was normalized with the average expression level in healthy donor livers using 2^-ΔΔCt^ method [13].

### 2.5. Statistical analyses

Continuous variables were compared by *t*-test or Mann-Whitney *U* test. The categorical variables were compared by chi-square Fisher’s test. Gene expression level correlation was analyzed by Pearson correlation analysis. *p* < 0.05 was considered statistically significant. 

## 3. Results

### 3.1. Cell adhesion molecules-related pathways in acute phase liver graft injury

Eleven sets of RNA-Seq data of liver grafts from recipients with (*n* = 6) or without (*n* = 5) post-transplant HCC recurrence were subjected to pathway enrichment analyses based on the use of differentially expressed genes between these two recipient groups. The top five pathways frequently altered in our studied condition are those related to steroid hormone biosynthesis, retinol metabolism, metabolism of xenobiotics by cytochrome P450, drug metabolism by cytochrome P450, and finally, cell adhesion molecules (Supplementary Table S4). Among these pathways, we focused on pathways related to cell adhesion molecules for further study, not only because of their diversified roles in liver tumorigenesis, but also because of their expression in immune cells important for post-transplant tumor recurrence [14,15,16].

### 3.2. HFE and CD274 are two cell adhesion molecules with differential involvement in acute phase liver graft injury

**Figure 2:**
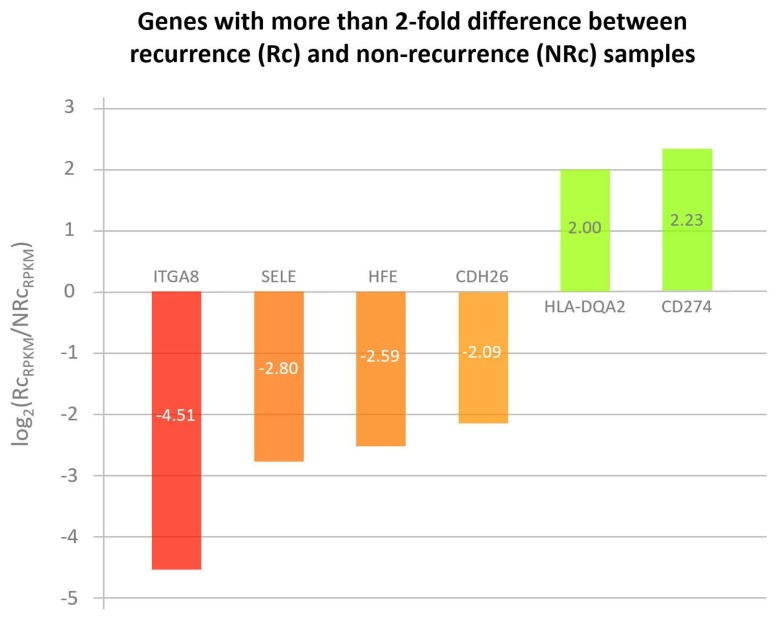
RNA-Seq result shows cell adhesion molecules that had more than two-fold gene expression level difference between recurrence and non-recurrence samples in acutely injured liver grafts. Six cell adhesion molecules with intragraft gene expression level difference of more than two-fold when recurrence samples were compared to non-recurrence ones. Four molecules (*ITGA8*, *SELE*, *HFE*, and *CDH26*) have been down-regulated in recurrence samples, whereas two molecules (*HLA-DQA2* and *CD274*) were found to have been up-regulated.

**Figure 3:**
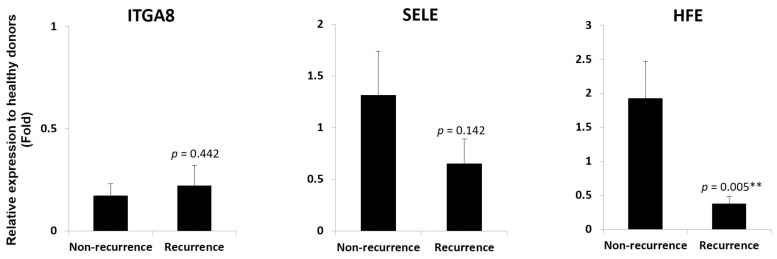
*HFE* down-regulation in liver grafts from recipients with post-transplant HCC recurrence. RT-qPCR result demonstrated a down-regulation of *HFE*, but not *ITGA8* and *SELE*, in liver grafts from recipients with post-transplant HCC recurrence. ** *p* < 0.01.

**Figure 4:**
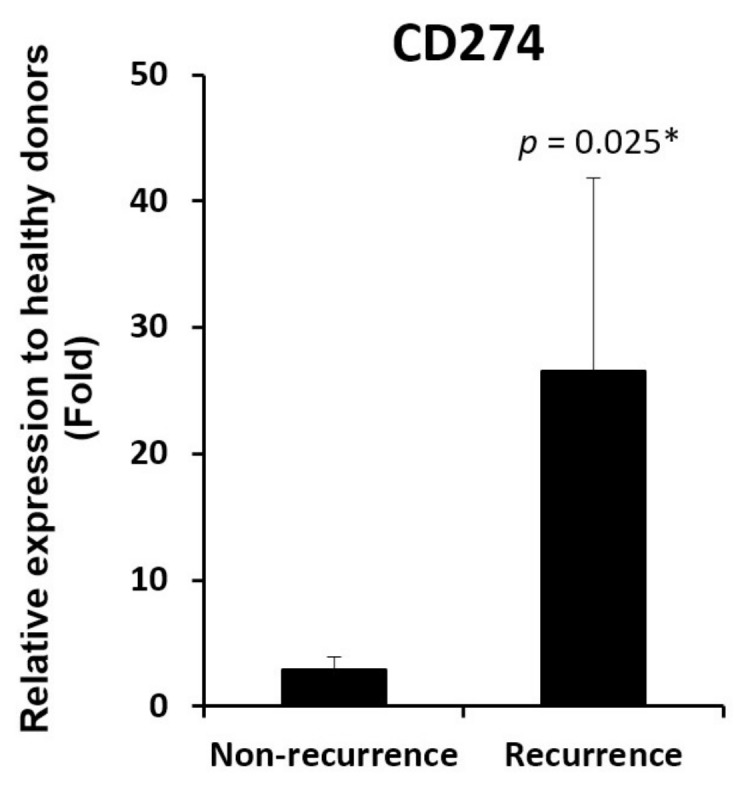
*CD274* up-regulation in liver grafts from recipients with post-transplant HCC recurrence. RT-qPCR result demonstrated an up-regulation of *CD274* in liver grafts from recipients with post-transplant HCC recurrence. * *p* < 0.05.

Cell adhesion molecules represent a broad class of membrane-associated molecules with diversified functions in pathophysiological processes, ranging from cell adhesion, inflammation, tissue injury, to tumorigenesis. In this category, seventy-five genes were differentially expressed in liver grafts in the recurrence group compared to the non-recurrence group (Supplementary Table S5), implicating their involvement in post-transplant HCC recurrence. Six genes with more than two-fold expression level difference between these two groups, i.e., *ITGA8*, *SELE*, *HFE*, *CDH26*, *HLA-DQA2*, and *CD274* (Figure 2 and highlighted in Supplementary Table S5), were subjected to RT-qPCR validation in a sample set of seven recurrences and seven non-recurrences. Among the four genes down-regulated in recurrence samples (*ITGA8*, *SELE*, *HFE*, and *CDH26*), only *HFE* maintained its down-regulation (Figure 3). The *CDH26* validation result was not shown due to its nearly undetectable expression level in our current experimental setting. For the two genes up-regulated in recurrence samples (*HLA-DQA2* and *CD274*), an up-regulation of *CD274* was maintained (Figure 4). No validation experiment was performed on *HLA-DQA2* due to its ubiquitous nature.

### 3.3. HFE- and CD274-related molecules in acutely injured liver grafts

**Figure 5:**
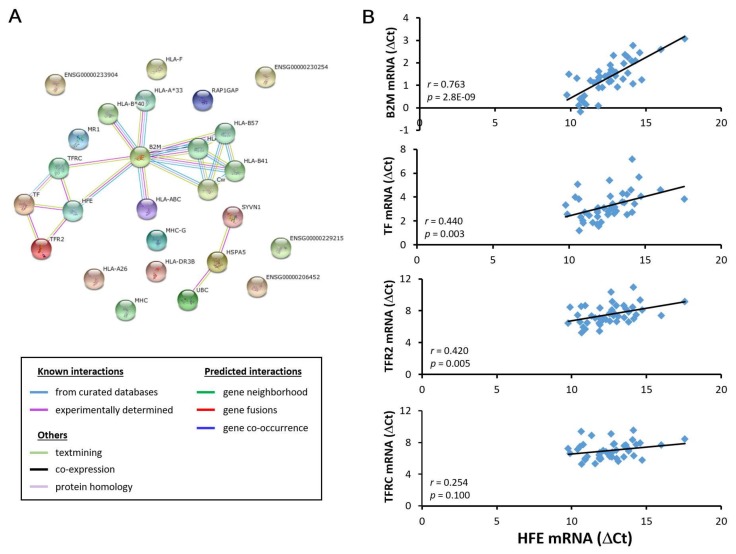
Correlation analysis of *HFE* and its related molecules in liver grafts from transplant recipients. (**A**) Bioinformatics analysis revealed *B2M*, *TF*, *TFR2* and *TFRC* as *HFE*-related molecules; (**B**) Among these molecules, *B2M*, *TF* and *TFR2*, but not *TFRC*, demonstrated a positive gene expression correlation with *HFE* in liver grafts from 43 transplant recipients using RT-qPCR.

**Figure 6:**
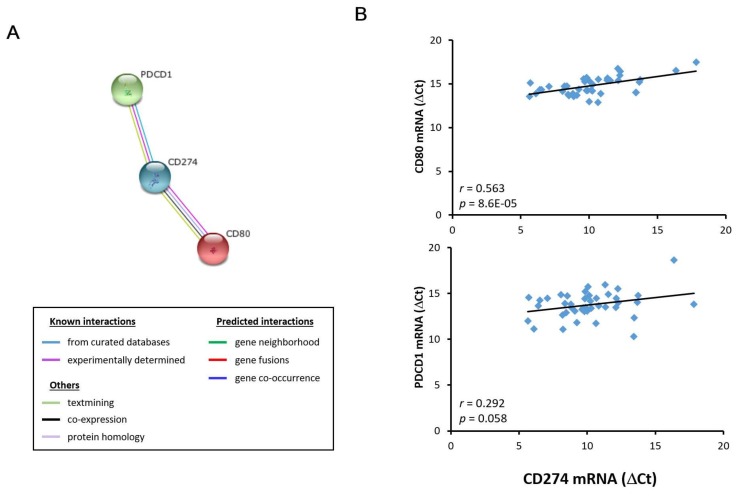
Correlation analysis of *CD274* and its related molecules in liver grafts from transplant recipients. (**A**) Bioinformatics analysis revealed that *CD80* and *PDCD1* were *CD274*-related molecules; (**B**) *CD80*, but not *PDCD1*, demonstrated a positive gene expression correlation with *CD274* in liver grafts from 43 transplant recipients using RT-qPCR.

STRING database result revealed four *HFE*-related molecules (*B2M*, *TF*, *TFR2*, and *TFRC*) (Figure 5A and Supplementary Table S6) and two *CD274*-related molecules (*CD80* and *PDCD1*) (Figure 6A and Supplementary Table S6). Their gene expression level correlation with HFE and CD274 was performed in liver grafts from 43 transplant recipients (6 recurrence and 37 non-recurrence) using RT-qPCR. Among the *HFE*-related molecules, *B2M*, *TF*, and *TFR2*, but not *TFRC*, demonstrated positive gene expression level correlation with *HFE* (Figure 5B). Among the *CD274*-related molecules, *CD80*, but not *PDCD1*, exhibited positive gene expression level correlation with *CD274* (Figure 6B). Collectively, our results revealed that the involvement of *HFE* and *CD274* and their related molecules in acute phase liver graft injury and their concurrent involvement in post-transplant tumor recurrence. 

## 4. Discussion

Our previous studies have identified that the acute phase liver graft injury is a key event leading to post-transplant HCC recurrence. In this study, we adopted a more comprehensive and unbiased approach using RNA sequencing to analyze differentially expressed genes in this acute phase event, with an aim to identify key players. The sequencing data revealed a subset of cell adhesion molecules differentially expressed in liver grafts from recipients with or without post-transplant tumor recurrence. Cell adhesion molecules belong to a class of cell surface molecules with diversified functions from adhesion, migration, to inflammation [16]. In liver transplantation, inflammation is initiated in the hallmark event of ischemia and reperfusion injury resulting from hepatic surgery. Massive recruitment of immune cells, which express various cell adhesion molecules, takes place during acute phase liver graft injury that can eventually lead to post-transplant tumor recurrence [17]. Certain cell adhesion molecules, e.g., MHC molecules [18], are involved in ischemia and reperfusion liver injury. In addition, our previous studies have demonstrated that certain molecules, e.g., aldose reductase and repressor and activator protein, are capable of regulating hepatic ischemia and reperfusion injury through their effects on inflammation [19,20]. Collectively, our and other findings have exemplified the key roles of cell adhesion molecules in inflammation, ischemia, and reperfusion liver graft injury, as well as post-transplant tumor recurrence. However, due to the small sample size that was used, findings that were derived from this study should be further validated in a separate cohort of large sample size. To minimize variations between patients, we tried to analyze specimens from patients that were selected based on the Milan criteria. 

*HFE* (hemochromatosis) is a cell adhesion molecule that was identified in this study to have down-regulated gene expression level in liver grafts from recipients with post-transplant HCC recurrence. It is an atypical MHC class I molecule with diversified cellular functions, such as iron homeostasis maintenance and immune function regulation [21,22]. Mutation of this gene can lead to iron overload, which is a predisposing factor for HCC [21,23]. Besides, two studies have reported the close link between hepatic iron overload and poor survival of liver transplant recipients [24,25]. In this study, we have also established a positive correlation in the intragraft gene expression of *HFE* and its related molecules (*B2M*/β2-microglobulin, *TF*/transferrin, and *TFR2*/transferrin receptor 2) in liver transplant recipients, for which these molecules are known iron metabolism regulators with close interaction with *HFE* [21,26]. Taken together, it is convincing to believe that *HFE* down-regulation in liver grafts, as observed in this study, can lead to iron overload and eventually post-transplant tumor recurrence. 

In contrary to *HFE*, we have demonstrated a high intragraft gene expression level of *CD274* from recipients with post-transplant tumor recurrence. *CD274*, also known as PD-L1, is expressed on immune cells, as well as non-hemopoietic cells [27]. In addition to its general immunoregulatory functions, *CD274* is also involved in tumorigenesis, as reflected by its high expression level in tumor tissues rather than in adjacent non-tumor tissues of various cancers [27,28]. The tumor-related function of *CD274* can also help to explain for its high intragraft gene expression level in transplant recipients with tumor recurrence as observed in this study. The tumor-inducing effect of *CD274* may also involve other immunoregulatory molecules, such as *CD80*, whose intragraft gene expression level correlated positively with *CD274* in transplant recipients as reported here. Like *CD274*, *CD80* is also found on immune cells and is involved in an array of immune pathways [29]. Indeed, both molecules are known to participate in transplantation immunity [30]. In view of these interesting observations from this and other studies, it is plausible that *CD274* may work with its related molecule, e.g., *CD80*, in triggering acute phase liver graft injury and post-transplant tumor recurrence.

Taken together, we have successfully used RNA sequencing to unravel the molecular landscape of acute phase liver graft injury that accounts for post-transplant tumor recurrence. Certain cell adhesion molecules, e.g., *HFE* and *CD274*, were found to have differential roles in our studied condition. These molecules have potential function as a prognostic marker for risk assessment to identify transplant recipients more prone to tumor recurrence and to guide them for prophylactic treatment for prevention. Apart from the preventive measure, the identified molecules can also form a basis for research on new treatment targets.

## Supplementary Materials

The following are available online at http://www.mdpi.com/2227-9059/6/2/41/s1.
