# Supplementary material for: Transcriptome Analysis of Acute Phase Liver Graft Injury in Liver Transplantation
Source: Biomedicines. 2018 Apr 6;6(2):41. doi: 10.3390/biomedicines6020041 (PMC6027418; doi:10.3390/biomedicines6020041)
Supplement: Supplementary file 1 [file biomedicines-06-00041-s001.pdf]

Table 1. Clinicopathological parameters of recurrence and non-recurrence groups.

Clinicopathological parameters	Recurrence group (N = 6)	Non-recurrence group (N = 37)	p value
Sex (number) [a]			
Male	5	30	1.000
Female	1	7	
Age (median) [b]	51 (41–57)	55 (30–67)	0.083
Type of liver transplantation [a]			
DDLT	1	2	0.37
LDLT	5	35	
Milan criteria [a]			
Within criteria	2	23	0.218
Beyond criteria	4	14	
Vascular permeation [a]			
No	3	24	0.655
Yes	3	13	
HBsAg before liver transplantation [a]			
Negative	0	6	0.571
Positive	6	31	
New TNM stage [a]			
Early stage (stage I or II)	5	36	0.262
Advanced stage (stage III or IV)	1	1	
AST level at Post-LT 24hr (u/l) (Mean Rank) [b]	36.33	19.68	0.003 **
ALT level at Post-LT 24hr (u/l) (Mean Rank) [b]	33.00	20.22	0.021 *

[a] statistical analysis was conducted by chi-square test (Fisher's exact); [b] statistical analysis was conducted by Mann-Whitney U test; DDTL, deceased donor liver transplant; LDLT, living donor liver transplant; * $p < 0.05$; ** $p < 0.005$

Table 2. Recurrence period and recurrence sites of HCC patients after liver transplantation.

Patient number	Recurrence period (months)	Recurrence sites
1	44	Liver
2	39	Lung
3	4	Lung, Bone, Gum
4	5	Lung, Brain
5	17.5	Lung
6	10.5	Lung, Liver, Brain

Table 3. qPCR primers.

Gene	Forward primer (5′ to 3′)	Reverse primer (5′ to 3′)
B2M	GATGAGTATGCCTGCCGTGT	TGCGGCATCTTCAAACCTCC
CD274	CCTGCAGGGCATTCCAGAAA	AGTGCAGCCAGGTCTAATTGT
CD80	CGCCTCTCTGAAGATTACCCA	TTCACAGCTTGCTGAAGAAAAA
HFE	CCTCTTCATGGGTGCCTCAG	TTTCACAGCCCAGGATGACC
ITGA8	AAAAGCAGACGGAAGTGGCT	AACCAATTCTTGCTGAGAATCCC
PDCD1	CAGTTCCAAACCCTGGTGGT	GGCTCCTATTGTCCCTCGTG
SELE	CCTGTGAAGCTCCCACTGAG	CCGTAAGCATTTCCGAAGCC
TF	CGGAAGCCGGTAGATGAATA	TGGTTGAGAAGCTCCCAGAT
TFR2	CCCAGAAGGTGACCAATGCT	TGTCCATACACTGCCTGCTG
TFRC	GGGATACCTTTCGTCCCTGC	ACCGGATGCTTCACATTTTGC
β-actin	CTCTTCCAGCCTTCCTTCCT	AGCACTGTGTTGGCGTACAG

Table 4. Pathways involved in acute phase liver graft injury.

Pathway	Average weight (recurrence/non-recurrence)	Pathway ID
Steroid hormone biosynthesis	0.945	ko00140
Retinol metabolism	0.945	ko00830
Metabolism of xenobiotics by cytochrome P450	0.942	ko00980
Drug metabolism—cytochrome P450	0.94	ko00982
Cell adhesion molecules (CAMs)	0.913	ko04514
Drug metabolism—other enzymes	0.866	ko00983
Amoebiasis	0.836	ko05146
African trypanosomiasis	0.817	ko05143
Caffeine metabolism	0.814	ko00232
Pathways in cancer	0.809	ko05200
Ascorbate and aldarate metabolism	0.785	ko00053
PPAR signaling pathway	0.784	ko03320
Dilated cardiomyopathy	0.779	ko05414
Malaria	0.78	ko05144
Bile secretion	0.776	ko04976
Vascular smooth muscle contraction	0.773	ko04270
Chagas disease (American trypanosomiasis)	0.769	ko05142
Hypertrophic cardiomyopathy (HCM)	0.765	ko05410
Ribosome biogenesis in eukaryotes	0.761	ko03008
Arachidonic acid metabolism	0.742	ko00590

Table 5. Cell adhesion molecules involved in acute phase liver graft injury.

Gene symbol	Gene name	Average value, $\log_2(R_{CRPKM}/NR_{CRPKM})$ (recurrence/non-recurrence)	Standard deviation
**ITGA8**	**Integrin subunit alpha 8**	−4.51	2.57
**SELE**	**Selectin E**	−2.8	2.07
**HFE**	**Hemochromatosis**	−2.59	0.86
**CDH26**	**Cadherin 26**	−2.09	1.12
CNTNAP2	Contactin associated protein like 2	−1.71	1.05
CD34	CD34 molecule	−1.68	1.1
ITGA9	Integrin subunit alpha 9	−1.56	1.14
BOC	BOC cell adhesion associated, oncogene regulated	−1.53	1.31
TMCC2	Transmembrane and coiled-coil domain family 2	−1.48	1.34
ITGAL	Integrin subunit alpha L	−1.43	0.76
NCAM1	Neural cell adhesion molecule 1	−1.36	0.85
MPZL3	Myelin protein zero like 3	−1.35	1.19
CLDN10	Claudin 10	−1.26	1.09
ITGB7	Integrin subunit beta 7	−1.11	0.68
CD58	CD58 molecule	−1.1	0.58

NFASC	Neurofascin	−1.1	1.04
JAM2	Junctional adhesion molecule 2	−1.02	0.81
PTPRF	Protein tyrosine phosphatase, receptor type F	−1	0.35
PTPRM	Protein tyrosine phosphatase, receptor type M	−0.99	0.66
HLA-DRB5	Major histocompatibility complex, class II, DR beta 5	−0.94	1.84
CADM3	Cell adhesion molecule 3	−0.93	1.73
CLDN16	Claudin 16	−0.92	1.04
WWC2	WW and C2 domain containing 2	−0.91	0.34
NLGN2	Neuroligin 2	−0.84	0.87
SRPX	Sushi repeat containing protein, X-linked	−0.83	0.65
NEGR1	Neuronal growth regulator 1	−0.81	1.38
CPNE2	Copine 2	−0.78	0.47
SELL	Selectin L	−0.74	2.04
SDC2	Syndecan 2	−0.74	0.73
CLDN14	Claudin 14	−0.71	1.19
CDH2	Cadherin 2	−0.68	0.66
CNTNAP1	Contactin associated protein 1	−0.66	1.18
AZGP1P1	Alpha-2-glycoprotein 1, zinc-binding pseudogene 1	−0.65	0.74
NRXN1	Neurexin 1	−0.55	1.19
CLDN11	Claudin 11	−0.55	0.77
CNTN1	Contactin 1	−0.41	1.18
NRXN3	Neurexin 3	−0.36	1.6
L1CAM	L1 cell adhesion molecule	−0.35	1.43
ITGB2	Integrin subunit beta 2	−0.35	1.05
ITGAM	Integrin subunit alpha M	−0.34	1.21
PTPRC	Protein tyrosine phosphatase, receptor type C	−0.32	1.47
ITGA4	Integrin subunit alpha 4	-0.31	1.62
CPNE5	Copine 5	−0.29	0.71
CD86	CD86 molecule	−0.29	1.02
CD4	CD4 molecule	−0.2	1.02
CDKN2B-AS1	CDKN2B antisense RNA 1	−0.16	1.32
JAM3	Junctional adhesion molecule 3	−0.15	0.61

SELP	Selectin P	−0.14	1.1
EMP1	Epithelial membrane protein 1	−0.11	0.9
SELPLG	Selectin P ligand	−0.1	1.26
CD40	CD40 molecule	−0.09	0.77
PDCD1	Programmed cell death 1	−0.04	3.14
MAG	Myelin associated glycoprotein	0.02	4.36
CDH1	Cadherin 1	0.06	0.91
FNDC1	Fibronectin type III domain containing 1	0.09	2.79
HLA-DOA	Major histocompatibility complex, class II, DO alpha	0.15	1.18
MPZ	Myelin protein zero	0.18	0.64
VCAN	Versican	0.23	1.42
CD22	CD22 molecule	0.29	2.7
HLA-F	Major histocompatibility complex, class I, F	0.44	1.33
CD226	CD226 molecule	0.46	1.3
HLA-DRB4	Major histocompatibility complex, class II, DR beta 4	0.58	4.58
CNTNAP3	Contactin associated protein like 3	0.72	1.77
MPZL1	Myelin protein zero like 1	0.72	0.3
CNTNAP3B	Contactin associated protein like 3B	0.76	1.14
CPNE6	Copine 6	0.79	4.73
CLDN1	Claudin 1	0.81	0.66
F11R	F11 receptor	0.96	0.46
PSORS1C3	Psoriasis susceptibility 1 candidate 3	0.96	4.14
PVR	Poliovirus receptor	1.07	0.6
CD276	CD276 molecule	1.53	0.58
HLA-DOB	Major histocompatibility complex, class II, DO beta	1.61	0.8
CLDN9	Claudin 9	1.91	1.7
**HLA-DQA2**	**Major histocompatibility complex, class II, DQ alpha 2**	2	1.67
**CD274**	**CD274 molecule**	2.23	1.46

* NRc: non-recurrence; R: recurrence.

Table 6. Differential expression of genes related to HFE and CD274.

Gene	$\log_2(Rc_{RPKM}/NRc_{RPKM})$	Gene	$\log_2(Rc_{RPKM}/NRc_{RPKM})$
		TFR2	−0.499
		TF	0.799
		TFRC	0.903
HFE	−2.59	*B2M*	0.002
		PDCD1	−0.110
CD274	2.23	*CD80*	0.167

* NRc: non-recurrence; R: recurrence
